# Sleep and mood disorders in women with dry eye disease

**DOI:** 10.1038/srep35276

**Published:** 2016-10-12

**Authors:** Masahiko Ayaki, Motoko Kawashima, Kazuno Negishi, Taishiro Kishimoto, Masaru Mimura, Kazuo Tsubota

**Affiliations:** 1 Department of Ophthalmology, Keio University School of Medicine, 35 Shinanomachi, Shinjuku, 1608582 Tokyo, Japan; 2 Shinseikai Hospital Eye Center, Imizu, Japan; 3 Psychiatry, Keio University, School of Medicine, Tokyo, Japan

## Abstract

The aim of the present study was to evaluate sleep and mood disorders in women aged 30–69 with dry eye disease (DED). All subjects underwent corneal examinations, with 890 completing a questionnaire regarding symptoms of DED and 213 completing both the Pittsburgh Sleep Quality Index (PSQI) and the Hospital Anxiety and Depression Scale (HADS) questionnaires. Subjects were then divided into three groups based on age (younger [30–45 years], perimenopausal [46–55 years], and older [56–69 years]), and comparisons were made among groups in subjects with and without DED. PSQI scores were significantly worse in subjects with (6.1 ± 2.9) than without (4.9 ± 2.7) DED (*P* = 0.003) and, in the younger group, HADS scores were worse in those with (13.2 ± 6.0) than without DED (9.7 ± 6.0) (*P* = 0.020). In contrast, there were no differences in mood indices between those with and without DED in the other groups. PSQI score was significantly correlated with HADS rather than ocular findings. In conclusion, sleep quality had deteriorated in women with DED. However, mood problems contributed more to sleep quality than ocular status, especially in those with DED in the younger group.

Dry eye disease (DED) is characterized by a variety of visual and non-visual symptoms, including tear deficiency, excessive tear evaporation, ocular surface inflammation, and corneoconjunctival epitheliopathy. DED is most prevalent in women of climacteric age^1^,^2^ and they suffer from low quality of life, which may be associated with the severity of DED^3^.

In a previous study we described poor sleep quality in DED^4^; ocular conditions may affect sleep by disturbing the ocular surface, causing pain and discomfort. Poor sleep quality has been correlated with both the presence of DED in high school students^5^ and a low Shirmer value in office workers^6^. Conversely, sleep deprivation could possibly reduce tear secretion^7^. If subjects with DED also have nocturnal lagophthalmos, their quality of sleep may deteriorate even further because of ocular irritation. Other studies have reported that subjects with Sjögren’s syndrome often have sleep apnea^8^ and that subjects with DED have a short sleep duration^9^. Poor sleep quality is directly associated with a shortened life expectancy and increased prevalence of various diseases, including diabetes and hypertension^10^,^11^.

Recent studies have reported that subjects with DED are depressed^12^,^13^,^14^,^15^,^16^,^17^, and depression is a common psychiatric problem in women that is closely associated with sleep disorders^18^,^19^,^20^. During the perimenopausal period, hormonal changes have marked effects on women’s health, including mental health, the endocrine system, obstructive sleep apnea, and the circulatory system^18^,^19^,^20^. Hormone-replacement therapy has been proposed for the treatment of DED and menopause-related disorders^21^,^22^,^23^. Although sleep quality, mood status, and DED could affect one another, to the best of our knowledge no study has evaluated sleep and mood disorders in women with DED over a broad age range.

Herein, we report on the results of clinical studies, including ocular examinations and a questionnaire-based survey, to explore the severity of sleep and mood disorders and their correlation with ocular status in those with and without DED, in women aged between 30 and 69 years.

## Methods

### Study institutions and Institutional Review Board approval

Female participants were consecutively recruited to the study between January 2014 and March 2016 from six general eye clinics in Japan. The Institutional Review Boards and Ethics Committees of Keio University School of Medicine, Shinseikai Toyama Hospital, and Komoro Kosei Hospital approved this study, and the study was performed in accordance with the principles of the Declaration of Helsinki. Informed consent was obtained from all participants.

### Ophthalmological examinations and treatments

All patients were examined by board-certified ophthalmologists specialized in corneal disorders. A diagnosis of DED was made according to the Japanese Dry Eye Society^24^,^25^, which classifies DED into definite DED (DDED), probable DED (PDED), and non-DED based on the presence of dry eye symptoms, tear abnormalities (Schirmer test ≤5 mm or tear BUT ≤5 s), maximum blinking interval (≤9 s)^26^, and superficial punctate keratoepitheliopathy (staining score ≥3).

Patients diagnosed with DDED or PDED were enrolled in the present study as subjects with DED. None of the patients had undergone any non-medical interventions, such as punctal plug insertion or punctal occlusion, or any surgical interventions. The eye drops prescribed for the treatment of DED contained hyaluronate, mucin secretagogue, and steroid, i.e., 0.1%/0.3% Hyalein^R^ (sodium hyaluronate; Santen Pharmaceutical Co. Ltd., Osaka, Japan), 0.1%/0.3% Tearbalance^R^ (sodium hyaluronate; Senju Pharmaceutical Co. Ltd., Osaka, Japan), Diquas^R^ (3% diquafosol sodium; Santen Pharmaceutical Co. Ltd), Mucosta^R^ (2% rebamipide, Otsuka Pharmaceutical, Co. Ltd., Tokyo, Japan), and 0.02%/0.1% Flumetholon^R^ (fluorometholon; Santen Pharmaceutical Co. Ltd).

### Patient interviews for DED-related symptoms

Outpatients were interviewed regarding symptoms related to DED. Questions determined the presence or absence of six common DED-related symptoms, namely dryness, irritation, pain, eye fatigue, blurring, and photophobia. The questions put to patients were selected from items in the Dry Eye Questionnaire Score (DEQS)^27^ based on the six most prevalent symptoms of DED patients visiting the Dry Eye Clinic of the Department of Ophthalmology, Keio University Hospital, in 2012.

### Questionnaire-based survey for sleep and mood disorders

Visitors to the eye clinics were invited to complete the Pittsburgh Sleep Quality Index (PSQI)^28^ and the Hospital Anxiety and Depression Scale (HADS)^29^ questionnaires. Each questionnaire was self-administered during a patient’s visit, with data collected from January through March 2014. Scores for each scale were calculated according to separate algorithms and the scores were then subjected to analysis. The cut-off point for PSQI was: a total score of 5 or greater is indicative of poor sleep quality. The cut-off points for the HADS were: 0–7 as normal; 8–10 as borderline abnormal (borderline case); 11–21 abnormal. The PSQI consists of seven subscales, whereas the HADS consists of depression (HADS-D) and anxiety (HADS-A) subscales. These questionnaires have been widely used for hospital-based surveys and are easy to answer, even by eye clinic visitors, because they do not contain difficult questions concerning severe psychiatric disease (e.g. suicide and hallucinations). Photophobia was evaluated using two representative questions from established questionnaires (i.e. the National Eye Institute Visual Function Questionnaire-25^30^). The photophobia score ranged from 100 (best) to 0 (worst).

### Patient evaluations for systemic comorbidities

The presence of systemic comorbidities was evaluated on the basis of brief interviews, chart review, and results from annual health check records, if available. Assessments of the use of sleep medications and antidepressants were included in the PSQI score.

### Inclusion and exclusion criteria for people with eye disease and systemic comorbidities

The present study recruited consecutive female patients, aged between 30 and 69 years with best corrected visual acuity equal to or better than 20/25 in both eyes, from participating clinics during the study period. Subjects with any corneal disease, cataract, fundus pathology, including macular diseases and diabetic retinopathy, and glaucoma, all of which may reduce visual function, were excluded from the study. In addition, patients with diagnosed psychiatric disease and taking specific psychiatric medications were excluded from the study.

### Statistical analysis

Subjects were divided into three groups based on age (younger [30–45 years], perimenopausal [46–55 years], and older [56–69 years]), and then into those with and without DED, served as control. Results regarding sleep and mood disorders were compared between those with and without DED in these different age groups; in Japan, 50 years is the mean of age at which menopause occurs^31^. Results of the Schirmer test and the tear BUT in the right eye were used for analysis.

Where appropriate, data are given as the mean ± SD. The HADS and PSQI scores in each group were calculated and compared among the specified groups. To identify which ophthalmic parameters were correlated with psychiatric problems (sleep and mood disorders) in DED, regression analysis was performed for those with DED, with psychiatric parameters as dependent variables and demographic (age) and ophthalmic parameters as independent variables. All analyses were performed using StatFlex (Atech, Osaka, Japan) with *P* < 0.05 considered significant.

## Results

In all, 1506 patients provided responses for the DED symptoms interview, with 890 of these aged between 30 and 69 years; 305 were aged 30–45 years (younger group), 255 were aged 46–55 years (perimenopausal group), and 330 were aged 56–69 years (older group). Within the younger, perimenopausal, and older groups, 161 (52.8%; mean age 38.2 years), 141 (55.3%; mean age 50.8 years), and 169 (51.2%; mean age 63.4 years), respectively, had DED. The PSQI and the HADS questionnaires were completed by consecutive 900 female patients in eye clinics. Of these, 106 and 107 female patients with and without DED, respectively, aged between 30 and 69 years of age met the criteria in the present study (Table 1). DED medication is also listed in Table 1.

**Figure 1:**
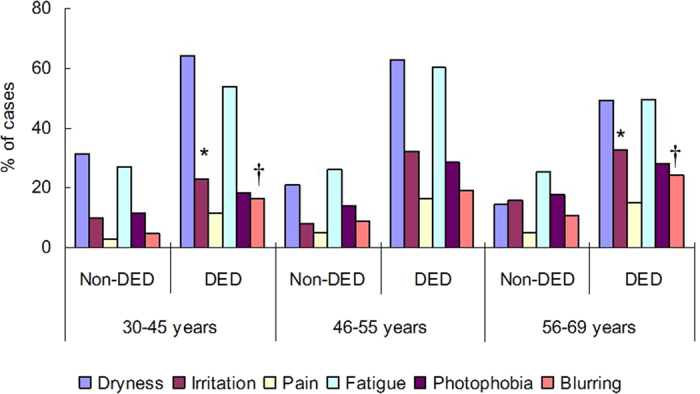
Prevalence of non-visual and visual dry eye disease (DED)-related symptoms in younger (30–45 years), perimenopausal (46–55 years), and older (56–69 years) women with and without DED. There was no significant difference in non-visual symptoms among the three age groups with DED. Regarding visual symptoms, photophobia (*P = 0.02, Chi-squared test) and blurring (^†^*P* = 0.02) were more prevalent in the older than younger group with DED. There was no significant difference in the prevalence of fatigue.

**Figure 2:**
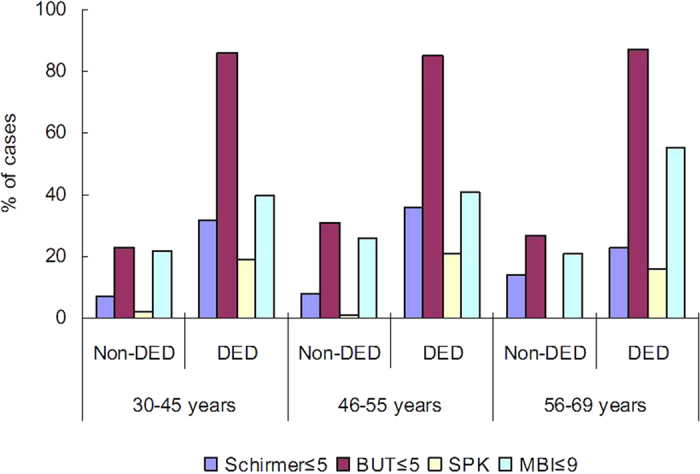
Ocular findings in younger (30–45 years), perimenopausal (46–55 years), and older (56–69 years) women with and without dry eye disease (DED). There were no significant differences in Schirmer test results, tear break-up time (BUT), superficial punctate keratitis (SPK), or maximum blinking interval (MBI) among the three age groups with DED.

Analysis of symptoms and signs revealed significant differences between the younger and older groups for the presence of photophobia and blurring (*P* = 0.03, *P* = 0.02, respectively, Chi-squared test), but not for the presence of dryness, eye fatigue, pain, or irritation (Fig. 1). Dryness and eye fatigue were the most prevalent symptoms of DED. Ocular surface examinations did not reveal any significant differences among three age groups (Fig. 2).

**Figure 3:**
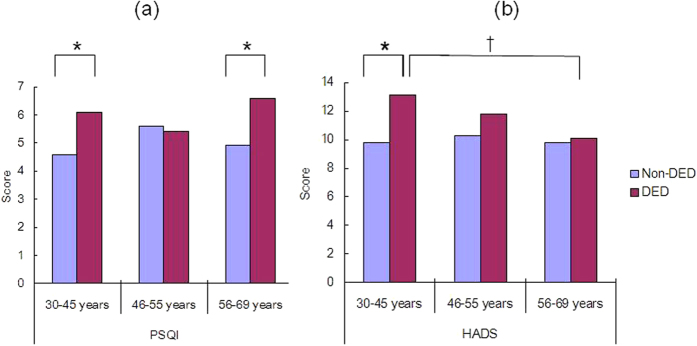
(**a**) Sleep and (**b**) mood disorders in younger (30–45 years), perimenopausal (46–55 years), and older (56–69 years) women with and without dry eye disease (DED), as determined by the Pittsburgh Sleep Quality Index (PSQI) and the Hospital Anxiety and Depression Scale (HADS) questionnaires, respectively. PSQI scores were significantly worse in women with than without DED in both the younger and older groups (*P = 0.001 and *P = 0.005, respectively). HADS scores were worse in younger subjects with than without DED (*P = 0.02), whereas there were no significant differences among the perimenopausal and other groups. It was also significantly worse in younger group than older group with DED (^†^*P* = 0.02). *P < 0.05 compared with subjects with DED in the same age group; ^†^*P* < 0.05 compared with older subjects with DED, Mann–Whitney *U*-test with Bonferroni correction.

**Figure 4:**
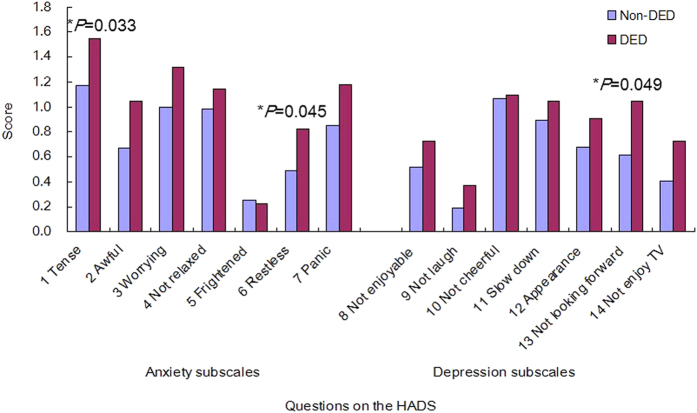
Hospital Anxiety and Depression Scale (HADS) subscores in women in the younger (30–45 years) group, with and without dry eye disease (DED). Younger women with DED had significantly worse scores for three items than women without DED (Mann–Whitney *U*-test). The first seven questions relate to the Anxiety subscale, whereas the last seven questions relate to the Depression subscale. Subjects were asked to respond Likert-type scale to the following questions on the HADS: 1, I feel tense; 2, I feel awful; 3, I am worried; 4, I can feel relaxed; 5, I get a sort of frightened feeling; 6, I feel restless; 7, I get sudden feelings of panic; 8, I still enjoy the things I used to enjoy; 9, I can laugh; 10, I feel cheerful; 11, I feel as if I am slowed down; 12, I have lost interest in my appearance; 13, I look forward with enjoyment to things; 14, I can enjoy TV. Questions are given in full in Zigmond and Snaith^29^.

The PSQI scores were significantly worse in the DED than non-DED group (*P* = 0.003, Mann–Whitney *U*-test), and this was most evident in the older DED group (*P* = 0.005) (Fig. 3a; Table 2). In the younger group, HADS scores were worse in the DED than non-DED group (*P* = 0.02), but there was no significant difference between those with and without DED in the other age groups (Fig. 3b). The PSQI subscales revealed significantly longer sleep latency (*P* = 0.049, Mann–Whitney *U*-test) and worse subjective sleep quality (*P* = 0.01) in DED compared with non-DED subjects in the younger group (Table 2). In the older group, subjects with DED had a shorter sleep duration (*P* = 0.001) and later bedtime (*P* = 0.000) than those without DED. Analysis of mood indices revealed that HADS scores (*P* = 0.02), anxiety subscores (*P* = 0.02), and depression subscores (*P* = 0.02) were significantly worse in those with than without DED in the younger group. In contrast, there were no significant differences in those with and without DED in the other age groups. Younger women with DED had significantly worse scores for some items on the HADS than women without DED (Mann–Whitney *U*-test; Fig. 4).

Regression analysis revealed that PSQI scores were closely correlated with mood indices including HADS score (*P* = 0.000), HADS-A score (*P* = 0.004), and HADS-D score (*P* = 0.000) (Table 3). Subjective sleep quality (*P* = 0.002, *P* = 0.021, *P* = 0.001, respectively) and sleep latency (*P* = 0.002, *P* = 0.012, *P* = 0.003, respectively) were significantly correlated with HADS, HADS-A, and HADS-D.

## Discussion

Sleep quality was significantly worse in women with than without DED. Mood disorders of depression and anxiety were major contributory factors, especially in the younger DED group. There was no indication in the results of regression analysis that ocular surface problems were directly associated with sleep quality.

Generally, depression worsens with age^18^,^19^; however, the results of the present study indicated that the depression score was worst in younger women with DED and, conversely, improved with age. In contrast, in women without DED, the results were comparable with previous reports, namely that sleep and mood decline gradually with age and are worst in the climacteric period. Labbé *et al*. reported that subjects with DED (≥40 years, mean age 64.8 years) were more depressive than those without DED, and that the level of depression was correlated with ocular symptoms, but not tear function, including results for the tear break-up time (BUT) and Schirmer test^13^. The results of large nation-wide survey using SF-36, validated and widely used measure for quality of life^32^, were supportive to both our findings and previous reports that scores of mental health was worst in young women aged 20–29, better with age, best in the age of 50–69, and decline after 70. Initially we hypothesized sleep and mood was worst in peri- or post-menopausal period and DED was an exacerbating factor for them and it was exactly the case with sleep quality in older group. In addition, DED affected sleep and mood most remarkably in younger group, and was not so much in peri-menopausal group, as shown in Fig. 3. Peri-menopausal symptoms depend on physical and psychosocial (personality, family, parents, colleagues) factors^33^ and it is reasonable that contributory factors to symptoms may vary at each life cycle. Further investigations are necessary to clarify the severity and interaction of DED and mood disorder in pre-menopausal women since many previous studies were for middle or older women.

The present study is the first to describe DED as a possible contributory factor to sleep disorders in women in midlife. It was evident in pre- and post-menopausal period rather than peri-menopausal. According to statistics from a coding database^12^, many disorders are often complicated with DED, including sleep apnea (odds ratio [OR] 2.46), depression (OR 1.92), and post-traumatic stress disorder (OR 1.92), and all of these could potentially exacerbate sleep quality in subjects with DED^11^. Insomnia, depression, and DED may be inter-related with each other in peri-menopausal period^34^.

In the present study, women in the older group reported more blurring and photophobia, which may be consequences of early cataract and presbyopia. This is another reason for the high prevalence of DED in the older population in the present study, because these conditions are supposedly linked with symptomatic DED, even though the visual acuity was better than 20/25 for women in the present study. There was no significant difference in corneal findings and the results of tear function tests between the younger and older groups. Older women with DED may suffer more from visual than non-visual symptoms.

In an attempt to compare DED patients with non-DED subjects without the possibility of confounding effects of systemic comorbidities, patients with glaucoma, cataract, visual impairment, and other ocular comorbidities that could affect sleep and mood were excluded from the study. Blindness, cataract^35^, and glaucoma^36^ can potentially exacerbate sleep and mood and recent studies^35^,^36^ have used pupillary examinations, polysomnography (electroencephalogram), and actigraphy in addition to questionnaires, and have revealed considerable associations between eye diseases and psychiatric status. Glaucoma is a very common eye disease that requires lifelong topical medication, which has known ocular surface toxicity^37^, and is often complicated by DED. Glaucoma patients are highly likely to experience sleep disorders^36^, and this is why glaucoma patients were excluded from the present study.

The present study has several limitations. The non-DED group consisted of eye clinic visitors who were diagnosed as having healthy vision and no ocular surface disease and normal biomicroscopic findings, although the Schirmer test and vital staining should have been performed to completely exclude subjects with DED from this group. Thus, the findings of the present study need to be confirmed using a comprehensive DED classification, such as the one proposed by the 2007 Dry Eye Workshop^38^. In addition, sleep and mood disorders should have been diagnosed precisely by qualified psychiatrists using polysomnography and actigraphy. Another limitation of the study is that it lacks sufficient consideration of systemic comorbidities and menopause status: because of time and space restrictions, we did not obtain a detailed medical history, but comorbidities and menopause status are important contributory factors to DED and sleep and mood disorders. This may have affected the results and further investigations that take comorbidities, socioeconomic status, education, and other possible confounding factors into account are needed to obtain a detailed understanding of the causal relationship between DED and psychiatric disorders.

In conclusion, sleep quality had deteriorated in women with DED. Mood problems contributed more to sleep quality than ocular status, especially in those with DED in the younger group. The findings of the present study may provide further information regarding mental health in women with DED.

## Additional Information

**How to cite this article**: Ayaki, M. *et al*. Sleep and mood disorders in women with dry eye disease. *Sci. Rep.* **6**, 35276; doi: 10.1038/srep35276 (2016).

## Figures and Tables

**Table 1:** Patient demographics in younger (30–45 years), perimenopausal (46–55 years), and older (56–69 years) women with and without dry eye disease (DED).

	Younger women			Perimenopausal women			Older women			All ages		
	Non-DED	DED	*P-*value	Non-DED	DED	*P-*value	Non-DED	DED	*P-*value	Non-DED	DED	*P-*value
No. patients	48	23		27	36		32	47	NS	107	106	
Age (years)												
Mean ± SD	37.6 ± 4.2	37.2 ± 4.2	NS	50.2 ± 3.2	50.2 ± 2.5	NS	62.6 ± 4.2	63.1 ± 4.2	NS	48.3 ± 11.4	10.7	0.001
Median	37	36		49	50		64	63		52	48	
Visual acuity (LogMAR)												
Worse eye	−0.08 ± 0.02	−0.05 ± 0.05	NS	−0.06 ± 0.04	−0.06 ± 0.04	NS	−0.04 ± 0.04	−0.04 ± 0.09	NS	−0.04 ± 0.03	−0.06 ± 0.03	NS
Better eye	−0.08 ± 0.03	−0.06 ± 0.04	NS	−0.06 ± 0.03	−0.06 ± 0.04	NS	−0.06 ± 0.04	−0.05 ± 0.04	NS	−0.06 ± 0.03	−0.07 ± 0.04	NS
High myopia (%)	4.2	17.3	NS	29.6	13.9	NS	18.8	2.1	NS	15.0	9.4	NS
Schirmer test (mm)	17.5 ± 10.9	11.2 ± 9.3	0.000	17.2 ± 9.2	9.9 ± 9.4	0.000	14.1 ± 8.7	9.3 ± 6.7	NS	16.0 ± 9.6	9.9 ± 8.0	0.000
Tear BUT (s)	5.5 ± 1.3	3.5 ± 1.4	0.000	5.3 ± 1.5	3.5 ± 1.3	0.000	5.5 ± 1.3	3.3 ± 1.3	NS	5.4 ± 1.4	3.4 ± 1.3	0.000
SPK (%)	2.1	18.6	0.000	0.9	21.1	0.000	0.0	16.0	NS	1.0	18.4	0.000
DED medication (%)												
Hyaluronate		95.7			58.3			70.2			71.7	
Mucin secretagogue		26.1			66.7			51.1			50.9	
Steroid		26.1			11.1			29.8			25.5	

Unless indicated otherwise, data are the mean ± SD. *P*-values were calculated using the Mann–Whitney *U*-test or Chi-squared test, as appropriate, and show comparisons for non-DED versus DED groups within a given age group.

Abbreviations: BUT, break-up time; SPK, superficial punctuate keratitis; NS, not significant.

**Table 2:** Sleep, mood, and related parameters in younger (30–45 years), perimenopausal (46–55 years), and older (56–69 years) women with and without dry eye disease (DED).

	Younger women			Perimenopausal women			Older women			All ages		
	Non-DED	DED	*P-*value	Non-DED	DED	*P-*value	Non-DED	DED	*P-*value	Non-DED	DED	*P-*value
PSQI global score	4.56 ± 2.62	6.09 ± 1.97	0.006	5.56 ± 3.17	5.35 ± 2.66	NS	4.91 ± 2.37	6.64 ± 3.38	0.005	4.92 ± 2.71	6.08 ± 2.92	0.003
Sleep efficacy score	0.23 ± 0.51	0.36 ± 0.65	NS	0.30 ± 0.72	0.25 ± 0.63	NS	0.13 ± 0.34	0.32 ± 0.75	NS	0.22 ± 0.53	0.22 ± 0.61	NS
Sleep disturbance score	0.69 ± 0.55	0.86 ± 0.35	NS	0.63 ± 0.49	0.78 ± 0.53	NS	0.94 ± 0.43	0.89 ± o.48	NS	0.75 ± 0.52	0.85 ± 0.47	NS
Pain	0.18 ± 0.44	0.20 ± 0.66	NS	0.11 ± 0.58	0.16 ± 0.51	NS	0.00 ± 0.00	0.17 ± 0.61	NS	0.08 ± 0.41	0.17 ± 0.58	NS
Bathroom^A^	0.25 ± 0.59	0.78 ± 1.04	0.02	0.56 ± 1.00	0.03 ± 1.10	NS	1.16 ± 1.05	1.30 ± 1.12	NS	0.60 ± 0.91	1.02 ± 1.15	0.003
Sleep medicine score	0.08 ± 0.45	0.23 ± 0.75	NS	0.44 ± 1.01	0.24 ± 0.76	NS	0.44 ± 1.05	0.77 ± 1.20	NS	0.28 ± 0.83	0.47 ± 1.01	NS
Subjective sleep score	1.08 ± 0.48	1.50 ± 0.69	0.01	1.31 ± 0.64	1.24 ± 0.81	NS	1.03 ± 0.55	1.36 ± 0.79	0.02	1.10 ± 0.58	1.35 ± 0.77	0.01
Daytime dysfunction score	0.63 ± 0.84	0.59 ± 0.73	NS	0.41 ± 0.80	0.78 ± 0.79	NS	0.50 ± 0.72	0.45 ± 0.62	NS	0.53 ± 0.79	0.59 ± 0.71	NS
Sleep duration (h:m)	6:25 ± 0:59	6:06 ± 1:15	0.06	5:45 ± 1:10	6:12 ± 0:56	NS	6:48 ± 0:56	6:02 ± 1:04	0.001	6:33 ± 1:05	6.11 ± 1:04	NS
Sleep latency (m)	16 ± 15	23 ± 16	0.049	19 ± 16	19 ± 23	NS	16 ± 14	24 ± 24	NS	16.0 ± 15.0	22.2 ± 22.8	NS
Bedtime (h:m)	23:56 ± 1:08	23:56 ± 1:27	NS	23:14 ± 1:11	24:06 ± 1:32	0.003	23:02 ± 0:44	24:04 ± 1:07	0.000	23:30 ± 1:08	23:56 ± 1:07	0.01
Photophobia score	89.6 ± 19.6	81.3 ± 17.4	0.01	84.7 ± 15.6	82.4 ± 21.1	NS	87.7 ± 15.6	84.6 ± 16.3	NS	87.8 ± 16.4	83.1 ± 18.3	0.052
HADS score	9.79 ± 5.82	13.18 ± 3.95	0.02	10.33 ± 4.81	11.83 ± 7.93	NS	9.78 ± 5.52	10.08 ± 5.49	NS	9.93 ± 5.4	11.36 ± 6.27	NS
HADS-A score	5.43 ± 3.25	7.27 ± 2.55	0.02	5.30 ± 2.81	6.13 ± 4.13	NS	5.13 ± 3.00	5.35 ± 2.83	NS	6.05 ± 3.04	5.31 ± 3.34	NS
HADS-D score	4.35 ± 3.30	5.91 ± 2.45	0.02	5.04 ± 2.62	5.71 ± 4.42	NS	4.66 ± 3.41	4.72 ± 3.26	NS	4.62 ± 3.16	5.32 ± 3.57	NS

Unless indicated otherwise, data are the mean ± SD. *P*-values were calculated using the Mann–Whitney *U*-test and are for comparisons between the non-DED and DED groups within a given age group.

Abbreviations: PSQI, Pittsburgh Sleep Quality Index; HADS, Hospital Anxiety and Depression Scale; HADS-A, HADS Anxiety subscore; HADS-D, HADS Depression subscore; NS, not significant.

^A^Sleep disturbance because of the need to use the bathroom at night.

**Table 3:** Regression analysis of psychiatric indices and ophthalmic parameters in women with dry eye disease (DED).

	Age	Schirmer test	Tear BUT	SPK	Severity of DED	Photophobia	HADS score	HADS–A score	HADS–D score
PSQI global score	0.155 (0.113)	0.161 (0.295)	−0.143 (0.228)	0.087 (0.377)	−0.030 (0.766)	−0.057 (0.569)	0.231 (0.000)	0.291 (0.004)	0.409 (0.000)
Sleep disturbance score	0.023 (0.811)	0.036 (0.815)	−0.135 (0.257)	0.119 (0.232)	−0.132 (0.181)	0.032 (0.748)	0.114 (0.263)	0.084 (0.415)	0.121 (0.233)
Bathroom^A^	0.200 (0.040)	−0.013 (0.930)	−0.184 (0.112)	0.037 (0.708)	−0.121 (0.226)	0.100 (0.304)	0.193 (0.056)	0.181 (0.071)	0.166 (0.093)
Pain	−0.023 (0.813)	0.283 (0.060)	0.176 (0.139)	0.162 (0.107)	0.118 (0.236)	−0.024 (0.815)	−0.021 (0.842)	−0.011 (0.914)	−0.024 (0.813)
Subjective sleep score	−0.046 (0.637)	0.088 (0.554)	0.009 (0.942)	0.110 (0.271)	−0.013 (0.898)	−0.023 (0.813)	0.315 (0.002)	0.235 (0.021)	0.330 (0.001)
Sleep latency	0.014 (0.884)	−0.074 (0.633)	−0.061 (0.610)	0.123 (0.219)	−0.028 (0.781)	0.066 (0.514)	0.320 (0.002)	0.256 (0.012)	0.297 (0.003)
Bedtime	−0.048 (0.623)	0.065 (0.651)	0.182 (0.119)	0.146 (0.141)	0.088 (0.380)	−0.094 (0.343)	0.018 (0.856)	−0.026 (0.804)	0.056 (0.584)
Photophobia	0.045 (0.651)	−0.087 (0.578)	0.183 (0.134)	0.174 (0.086)	−0.181 (0.073)	—	−0.044 (0.660)	−0.092 (0.370)	0.010 (0.921)
HADS score	−0.199 (0.045)	0.071 (0.656)	−0.008 (0.948)	0.018 (0.858)	−0.006 (0.953)	—	—	0.892 (0.000)	0.923 (0.000)
HADS-A score	−0.221 (0.025)	−0.039 (0.811)	−0.023 (0.850)	−0.040 (0.695)	−0.099 (0.328)	—	—	—	0.625 (0.000)
HADS-D score	−0.141 (0.157)	0.135 (0.377)	0.006 (0.958)	0.068 (0.500)	0.080 (0.423)	—	—	—	—

Data show β values, with *P*-values in parentheses. All results were adjusted for age.

PSQI, Pittsburgh Sleep Quality Index; HADS, Hospital Anxiety and Depression Scale; HADS-A, HADS Anxiety subscale score; HADS-D, HADS Depression subscale score; BUT, break-up time; SPK, superficial punctuate keratitis.

^A^Sleep disturbance because of the need to use the bathroom at night.
